# Modelling physiological and pathological conditions to study pericyte biology in brain function and dysfunction

**DOI:** 10.1186/s12868-018-0405-4

**Published:** 2018-02-22

**Authors:** Justin Rustenhoven, Leon C. Smyth, Deidre Jansson, Patrick Schweder, Miranda Aalderink, Emma L. Scotter, Edward W. Mee, Richard L. M. Faull, Thomas I.-H. Park, Mike Dragunow

**Affiliations:** 1 Centre for Brain Research, Faculty of Medical and Health Sciences, University of Auckland, 85 Park Rd, Grafton, Auckland, 1023 New Zealand; 2 Department of Pharmacology and Clinical Pharmacology, Faculty of Medical and Health Sciences, University of Auckland, 85 Park Rd, Grafton, Auckland, 1023 New Zealand; 3 Auckland City Hospital, Auckland, New Zealand; 4 Department of Anatomy and Medical Imagining, Faculty of Medical and Health Sciences, University of Auckland, 85 Park Rd, Grafton, Auckland, 1023 New Zealand; 5 Department of Pharmacology and Clinical Pharmacology, University of Auckland, Private Bag 92019, Auckland, 1142 New Zealand

**Keywords:** Inflammation, Phagocytosis, Migration, Proliferation, Growth factor, Blood–brain barrier

## Abstract

**Background:**

Brain pericytes ensheathe the endothelium and contribute to formation and maintenance of the blood–brain-barrier. Additionally, pericytes are involved in several aspects of the CNS immune response including scarring, adhesion molecule expression, chemokine secretion, and phagocytosis. In vitro cultures are routinely used to investigate these functions of brain pericytes, however, these are highly plastic cells and can display differing phenotypes and functional responses depending on their culture conditions. Here we sought to investigate how two commonly used culture media, high serum containing DMEM/F12 and low serum containing Pericyte Medium (ScienCell), altered the phenotype of human brain pericytes and neuroinflammatory responses.

**Methods:**

Pericytes were isolated from adult human brain biopsy tissue and cultured in DMEM/F12 (D-pericytes) or Pericyte Medium (P-pericytes). Immunocytochemistry, qRT-PCR, and EdU incorporation were used to determine how this altered their basal phenotype, including the expression of pericyte markers, proliferation, and cell morphology. To determine whether culture media altered the inflammatory response in human brain pericytes, immunocytochemistry, qRT-PCR, cytometric bead arrays, and flow cytometry were used to investigate transcription factor induction, chemokine secretion, adhesion molecule expression, migration, phagocytosis, and response to inflammatory-related growth factors.

**Results:**

P-pericytes displayed elevated proliferation and a distinct bipolar morphology compared to D-pericytes. Additionally, P-pericytes displayed lower expression of pericyte-associated markers NG2, PDGFRβ, and fibronectin, with notably lower αSMA, CD146, P4H and desmin, and higher Col-IV expression. Nuclear NF-kB translocation in response to IL-1β stimulation was observed in both cultures, however, P-pericytes displayed elevated expression of the transcription factor C/EBPδ, and lower expression of the adhesion molecule ICAM-1. P-pericytes displayed elevated phagocytic and migratory ability. Both cultures responded similarly to stimulation by the growth factors TGFβ_1_ and PDGF-BB.

**Conclusions:**

Despite differences in their phenotype and magnitude of response, both P-pericytes and D-pericytes responded similarly to all examined functions, indicating that the neuroinflammatory phenotype of these cells is robust to culture conditions.

**Electronic supplementary material:**

The online version of this article (10.1186/s12868-018-0405-4) contains supplementary material, which is available to authorized users.

## Background

The adult human brain contributes about 2% of an individual’s total body weight, yet receives 15–20% of the total blood supply. To meet these enormous energy demands, the brain contains an extensive vascular network. Brain vasculature has developed to include a highly selective permeability barrier termed the blood–brain barrier (BBB). This barrier is essential in segregating the central nervous system (CNS) from circulating blood and allows for the passage of numerous factors essential for correct cerebral functioning, whilst preventing the entry of potentially neurotoxic substances [1].

The BBB is comprised of brain endothelial cells strongly linked by tight junctions. In brain capillaries, these endothelial cells are ensheathed by pericytes embedded in the basement membrane. Astrocytic projections, termed end-feet, also contact brain vasculature, providing biochemical support. Together with neurons and perivascular microglia, this structure is collectively termed the neurovascular unit [2].

Brain pericytes are situated at a unique interface between the brain and periphery, allowing them to regulate multiple aspects of cerebral functioning. Pericytes are required for BBB formation during embryogenesis and pericyte coverage of vessels determines relative vascular permeability [3]. Due to their close association with the vasculature, pericytes can also contribute to angiogenesis through paracrine endothelial cell signalling [4] and controversially may modulate vasoconstriction [5–7].

Pericytes can also contribute to the formation of the basement membrane through the secretion of extracellular matrix (ECM) proteins [8]. After injury to the CNS, the ECM-secreting properties and migratory potential of pericytes allow them to participate in cerebral scarring [9]. Pericytes can also aid leucocyte extravasation through monocyte chemoattractant protein-1 (MCP-1) directed chemotaxis [10] and intercellular adhesion molecule-1 (ICAM-1) mediated adhesion and transmigration [11, 12]. Lastly, pericytes act as macrophages in the brain and demonstrate phagocytic ability, potentially aiding the cerebral clearance of foreign matter [13–15].

The multi-functional capability of pericytes may be partially explained by their highly plastic nature. Pericytes derived from peripheral tissues can differentiate into a range of cell types from mesodermal lineages including osteoblasts [16], chondrocytes [17], adipocytes [18] and myofibroblasts [19]. Indeed, pericytes are often considered a source of mesenchymal stem cells, to which they show very similar cell surface marker and gene expression profiles [20, 21]. However, forebrain pericytes originate from both the mesoderm and neuroectodermal-derived neural crest cells, suggesting that the mesenchymal nature of pericytes in the brain may be distinct from that of peripheral tissues [8]. In support of this, CNS pericytes can differentiate to express markers of neurons/astrocytes [22–24], microglia [25] and scar-forming myofibroblasts [9].

In vivo, pericytes can be identified by their anatomical location and unique bump-on-a-log morphology which appears as round nuclei protruding from a cylindrical vessel [26]. Identification of pericytes in vitro is more difficult and requires the use of pericyte-specific antigens. Several markers have been used to identify pericytes, including alpha-smooth muscle actin (αSMA), platelet-derived growth factor receptor-beta (PDGFRβ) and neural glial antigen-2 (NG2) [27, 28]. Importantly, none of these are specific for pericytes but identify a spectrum of cells including mesenchymal stem cells, pericytes, vascular smooth muscle cells and myofibroblasts [27, 28]. This uncertainty makes the characterisation of true capillary-derived pericyte cultures difficult and indeed phenotypic discrepancies exist between in vitro pericyte cultures from different research groups.

Differentiation and phenotypic changes in cells, both in vivo and in vitro, are highly regulated by growth factors. With respect to pericytes, transforming growth factor-beta1 (TGFβ_1_) and platelet-derived growth factor subunit B (PDGF-BB) are the most widely studied and drastically alter functional responses of pericytes including survival, proliferation, migration, differentiation, and scarring [8, 28, 29]. In vitro, growth factors are often provided at unknown amounts through the use of serum [e.g., fetal bovine serum (FBS)], or at specific concentrations in defined culture media. With regards to brain pericyte culture and analyses of functional outcomes, huge discrepancies exist in culture media ranging from defined Pericyte Medium containing low serum (2%) with additional (propriety) growth factors, to Dulbecco’s Modified Eagle’s Medium (DMEM; Additional file 1: Table S1) containing high serum (10–20%) [10, 13, 14, 20, 30–36]. Admittedly, variations in culture conditions extend far beyond media constituents including differences in donor species, age, and isolation procedure (Additional file 1: Table S1). Due to their highly plastic nature, it is conceivable that pericytes grown under different conditions adopt dissimilar phenotypes. Indeed pericytes cultured in a stem cell medium displayed distinct morphology and a greater differentiation potential than those grown in a defined Pericyte Medium [37].

DMEM containing 10–20% FBS and defined Pericyte Medium (ScienCell) have been used in the majority of recent in vitro pericyte-related studies. As such, we sought to identify how these distinct culture media influenced the phenotype of pericytes derived from the adult human and several functional responses including proliferation, inflammation, phagocytosis, migration, and growth factor response.

## Methods

### Tissue source

Human middle temporal gyrus (MTG) brain tissue was obtained, with informed consent, from surgeries of patients with drug-resistant temporal lobe epilepsy. All specimens were collected with written patient consent. All protocols used in this study were approved by the Northern Regional Ethics Committee (New Zealand) and all methods were carried out in accordance with the approved guidelines.

### Mixed glial cultures isolated from human brain tissue

Mixed glial cultures containing microglia, astrocytes, and pericytes were isolated from adult human brain tissue as described previously with minor modifications [38]. Approximately 2 g of tissue was washed in Hanks balanced salt solution (HBSS; Ca^2+^ and Mg^2+^ free; Gibco, CA, USA), the meninges and visible blood vessels were removed and tissue was diced into pieces approximately 1 mm^3^. Tissue was then added to 10 mL/g enzyme dissociation mix (10 U/mL DNase (Invitrogen, CA, USA) and 2.5 U/mL papain [Worthington, NJ, USA) in Hibernate-A medium (Gibco)] for 15 min at 37 °C with gentle rotation, triturated to aid digestion and incubated for a further 15 min. The tissue was further triturated and transferred to a new tube containing an equal volume of Dulbecco’s Modified Eagle Medium: Nutrient mixture F-12 (DMEM/F12; Gibco) supplemented with 10% fetal bovine serum (FBS; Moregate, Australia) and 1% penicillin–streptomycin–glutamine (PSG; Gibco). The cell suspension was passed through a 100 µm nylon cell strainer (Becton Dickenson, NJ, USA), centrifuged at 160×*g* for 10 min and the pellet resuspended in DMEM/F12. The resulting single cell suspension was split over multiple T75 tissue culture flasks (2 g tissue per three flasks; Nunc, Denmark) and incubated overnight at 37 °C with 95% air/5%CO_2_. The following day, media from the flask containing unattached cells and debris was removed and centrifuged at 160×*g* for 5 min. The resulting pellet was resuspended in DMEM/F12, 10% FBS, and 1% PSG and added back to the flask. On day three, media containing unattached cells or debris was removed and discarded. Cells were grown until confluent (1–2 weeks) in DMEM/F12, 10% FBS, and 1% PSG at which point they were harvested with 0.25% Trypsin-1 mM ethylenediaminetetraacetic acid (EDTA; Gibco) and gentle scraping using a rubber scraper (Falcon, MA, USA). Cells were cultured up to passage nine with early passages (2–3) containing microglia, astrocytes, and pericytes, whilst latter passages (4–9) contained only pericytes. Detailed characterisation of both early and late passage cultures has been performed previously [10, 39]. Briefly, late passage cultures were positive for pericyte markers αSMA, PDGFRβ, and NG2 and showed no expression of the astroglial marker GFAP or microglial markers PU.1 and CD45. Although a number of markers were used to characterise the pericyte cultures, no pericyte-specific antigen currently exists so it is possible that these cultures contain other fibroblast-like cells. For simplicity however, late passage cultures (4–9) will be referred to as pericytes. The relative proportions of microglia, astrocytes, and pericytes in these cultures at different passages has also been quantified. In general (there is some variation between different donors), ~ 10% CD45-positive microglia, ~ 5% GFAP-positive astrocytes, and 85% PDGFRβ-positive pericytes were observed in passage two cultures [40]. Passage five and above cultures contain ~ 100% pericytes and thus were used for all pericyte experiments.

### Cell plating for media experiments

Cells were harvested with 0.25% trypsin-ethylenediaminetetraacetic acid (EDTA; Gibco, CA, USA) and viable cells counted based on trypan blue exclusion (Gibco). Cells were plated in either DMEM/F12 supplemented with 10% FBS and 1% PSG or a defined Pericyte Medium (ScienCell; CA, USA; Cat # 1201) at a density of 2500 cells/well for 96 well plates, 25,000 cells/well for 24 well plates or 100,000 cells/well in 6 well plates (Nunclon Delta surface, Nunc, Denmark). Cells were grown for 5–14 days in this media with media changes every 2 days before being utilised for experiments. Henceforth, cells will be referred to as either DMEM/F12-cultured brain pericytes (D-pericytes) or Pericyte Medium cultured-brain pericytes (P-pericytes).

### Cytokine and growth factor treatments

To induce inflammatory responses, cells were treated with 10 ng/mL interleukin-1 beta (IL-1β; Peprotech, NJ, USA), or vehicle [0.0001% bovine serum albumin (BSA) in phosphate buffered saline (PBS)] for indicated times (0–24 h). To investigate the effect of growth factors on SMAD2/3 signalling and platelet-derived growth factor receptor beta (PDGFRβ) internalisation 10 ng/mL TGFβ_1_ (Peprotech, NJ, USA; vehicle 1 µM citric acid, pH 3 with 0.0001% BSA), or 10–100 ng/mL PDGF-BB (Peprotech; vehicle 4 µM hydrochloric acid with 0.0001% BSA) or vehicle was added for 0–2 h. Cytokines and growth factors were added by a 1:100 dilution of a 100× stock solution. To control for different growth factor vehicles, both TGFβ_1_ and PDGF-BB vehicles were added for vehicle control and the relevant vehicle was added in the absence of each growth factor.

### Collection of conditioned media and cytokine measurement by cytometric bead array

Conditioned media was collected from cells grown in 96-well plates. Media was spun at 160×*g* for 5 min to collect possible cells and debris. Supernatant was obtained and stored at − 20 °C. The concentration of cytokines (Additional file 2: Table S2) was measured using cytometric bead array (CBA; BD Biosciences, CA, USA) as described previously [41]. CBA samples were run on an Accuri C6 flow-cytometer (BD Biosciences, CA, USA). Data was analysed using FCAP-array software (version 3.1; BD Biosciences, CA, USA) to convert fluorescent intensity values to concentrations using a 10-point standard curve.

### Immunocytochemistry

Cells were fixed in 4% paraformaldehyde (PFA) for 15 min and washed in PBS with 0.1% triton X-100 (PBS-T). Cells were incubated with primary antibodies (Additional file 3: Table S3) overnight at 4 °C in immunobuffer containing 1% goat serum, 0.2% triton X-100 and 0.04% thiomersal in PBS. Cells were washed in PBS-T and incubated with appropriate anti-species fluorescently conjugated secondary antibodies overnight at 4 °C. Cells were washed again and incubated with Hoechst 33258 (Sigma-Aldrich, MO, USA) for 20 min. Images were acquired using the automated fluorescence microscope ImageXpress^®^ Micro XLS (Version 5.3.0.1, Molecular Devices, CA, USA). Quantitative analysis of intensity measures and positively stained cells was performed using the cell scoring and integrated morphometry analysis modules on MetaXpress^®^ software (Version 5.3.0.1, Molecular Devices). Roughly 500–1000 cells were scored per well with multiple wells (at least three) analysed per sample.

### EdU proliferation assay

Cells were grown for 5 days in either DMEM/F12 or defined Pericyte Medium and 10 µM 5-Ethyl-2′-deoxyuridine (EdU) was added for an additional 48 h. Cells were fixed and EdU was visualised via the Click-iT^®^ Plus EdU Alexa Fluor^®^ 647 Imaging Kit as per manufacturer’s instructions (Molecular Probes, OR, USA). Nuclei were counterstained with Hoechst 33258 and imaged on the ImageXpress^®^ Micro XLS microscope. The percentage of EdU-positive cells was determined using the Cell Scoring assay on MetaXpress^®^ software.

### Real time quantitative reverse transcriptase PCR

Cells were washed in PBS and RNA extraction and purification was performed using the RNeasy^®^ mini kit (Qiagen, Netherlands) as per manufacturer’s instructions. RNA was treated with DNase (1 µg DNase/1 µg RNA) using the RQ1 RNase-free DNase kit (Promega, WI, USA) and cDNA made using the Superscript^®^ III First-Strand Synthesis kit (Life Technologies, CA, USA). Quantitative real-time PCR was performed using Platinum^®^ SYBR^®^ Green qPCR SuperMix-UDG with Rox (Life Technologies, CA, USA) on a 7900HT Fast Real-Time PCR system (Applied Biosystems, Life Technologies, CA, USA). Standard curves were run for all primers used and efficiencies were all 100 ± 10% (Additional file 4: Table S4). Relative gene expression analysis was performed using the 2^−ΔΔCt^ method to the housekeeping gene glyceraldehyde 3-phosphate dehydrogenase (*GAPDH*).

### Phagocytosis assay

To evaluate phagocytosis by fluorescent microscopy, Fluoresbrite^®^ YG Carboxylate microspheres of 1–2 µm diameter (1:1000 dilution) was added to cells and centrifuged for 5 min at 160×*g*. Cells were then incubated with beads for 24 h at 37 °C, 5% CO_2_. At completion cells were washed twice with PBS to remove un-phagocytosed beads and fixed in 4% PFA as per immunocytochemistry. Nuclear staining was visualised by a 30 min incubation with DRAQ5 (Biostatus, UK). Images were obtained using the ImageXpress^®^ Micro XLS microscope and the percentage of phagocytic cells determined using the Cell Scoring module on MetaXpress^®^ software.

To evaluate phagocytosis by flow cytometry, Fluoresbrite^®^ YG Carboxylate microspheres of 1–2 µm diameter (1:1000 dilution) was added to cells and centrifuged for 5 min at 160×*g*. Cells were then incubated with beads for 2 h at 37 °C, 5% CO_2_. At completion, cells were washed twice with PBS and 0.25% trypsin–EDTA was added to remove beads bound to the cell surface and bring cells into suspension. Selected samples were incubated for 15 min on ice with 5 µL of 7-aminoactinomycin D (7-AAD; BD Biosciences, CA, USA) to assess viability. Samples were run on an Accuri C6 Flow Cytometer and viable cells gated based on forward scatter, side scatter, and 7-AAD exclusion. Mean fluorescent intensity (MFI) of the live cells was detected, indicative of the quantity of beads internalised.

### Flow cytometry

Flow cytometry was utilised to determine cell surface PDGFRβ as an indicator of internalisation following PDGF-BB stimulation. Cells were cultured in a 12-well plate and treated with vehicle (4 µM hydrochloric acid with 0.0001% BSA) or 10–100 ng/mL PDGF-BB for 30 min at 37 °C 5% CO_2_, the media was removed and cells were washed in PBS. Cells were brought into suspension by a 5 min incubation at 37 °C 5% CO_2_ with 300 µL of StemPro^®^ Accutase^®^ (Gibco), 700 µL of DMEM/F12 or Pericyte Medium was added and gently triturated. Cells were centrifuged at 160×*g* for 5 min, the supernatant was discarded, and the cell pellet was resuspended in 100 µL of flow cytometry buffer (1% FBS in PBS), 20 µL of PDGFRβ-PE (BD Biosciences) or isotype control (BD Biosciences), and 5 µL of 7-AAD. Samples were incubated on ice for 15 min in the dark, 1 mL of flow cytometry buffer was added and samples were centrifuged for 5 min at 160×*g*. The supernatant was discarded and samples were resuspended by gentle vortexing. Viable cells were gated based on forward scatter, side scatter, and 7-AAD exclusion.

### Scratch migration assay

To examine the ability of pericytes to migrate into a scratch wound, cells were grown until a ~ 100% confluent monolayer was formed. This took between 7 and 14 days when plated at 2500 cells/well in a 96-well plate. The cell monolayer was scratched down the centre of the well with a sterile 200 µL pipette tip and thoroughly washed with PBS to remove detached cells. Cells were cultured for a further 48 h to allow for migration to occur. Cells were fixed in 4% PFA for 15 min and stained with Coomassie Brilliant Blue R250 (0.25% in 10% glacial acetic acid, 45% methanol; Gibco, CA, USA) for 30 min, before removing this solution and allowing to air dry. Wells were acquired at 4× magnification using brightfield imaging (ImageXpress^®^ Micro XLS) and cells were identified by an inclusive threshold on MetaXpress^®^ software. The gap area (percentage of site imaged) was quantified by (total area-inclusive area)/total area.

### Statistical analysis

Unless otherwise stated, all experiments were performed at least three independent times on tissue from three different donors. Statistical analysis was carried out using an unpaired Student’s *t* test or a Two-Way ANOVA with Bonferroni post-test (GraphPad Prism 5.02). For statistical analysis of qRT-PCR data, ΔCt values were used.

## Results

### Culture media alters pericyte phenotype and proliferation

**Fig. 1:**
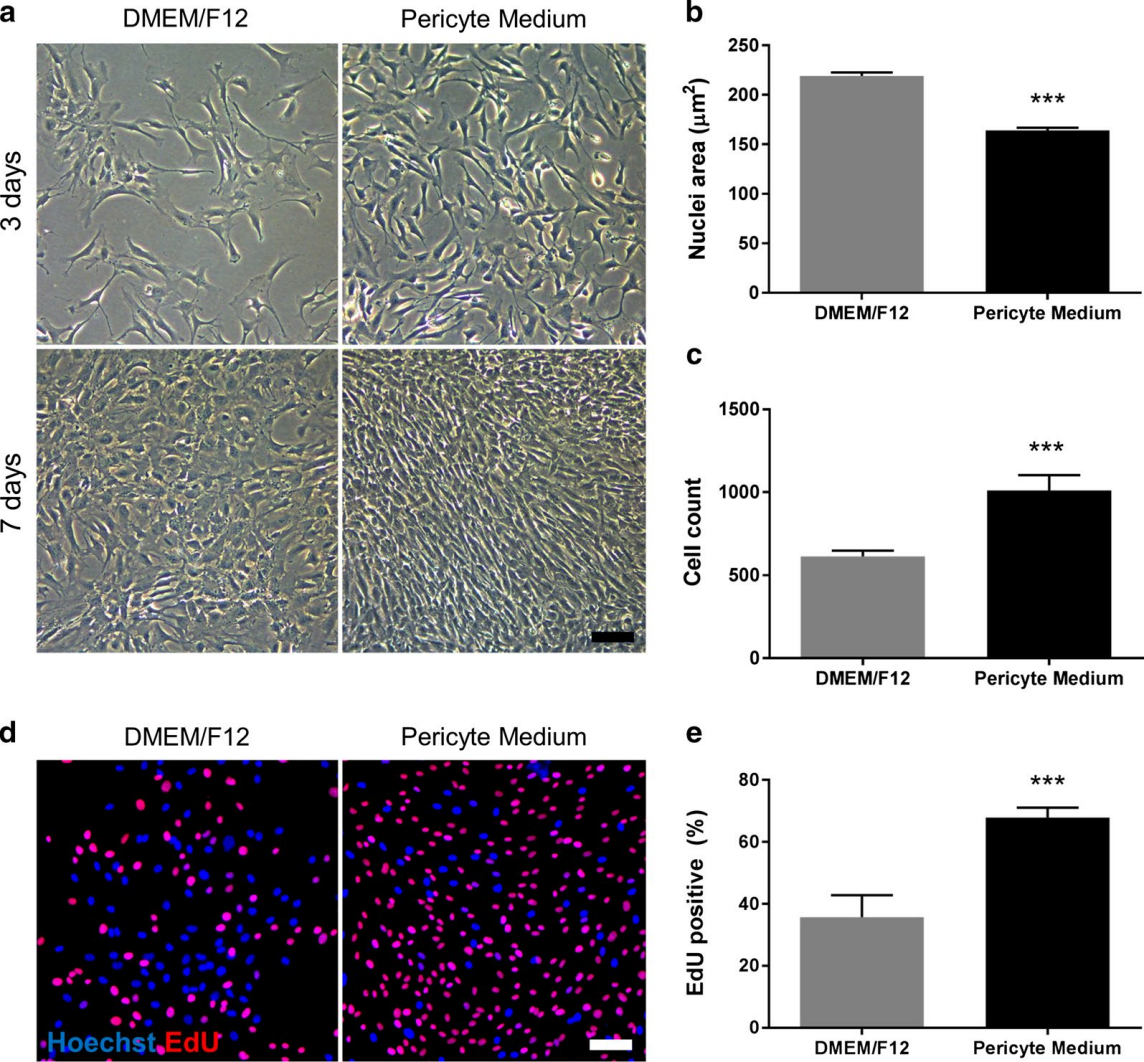
Culture media alters pericyte phenotype and proliferation. Human brain pericytes were plated at a low density and allowed to proliferate in either DMEM/F12 with 10% FBS or Pericyte Medium. Brightfield imaging of pericyte morphology was performed 3 and 7 days post plating (**a**). Pericytes were then fixed and the nuclei stained with Hoechst. The average nuclei area (**b**) and the total cell count (**c**) were determined by automated image analysis. Five days after plating, 10 µM EdU was added to select wells for an additional 48 h. Cells were fixed and nuclei counterstained with Hoechst (**d**). The percentage of EdU positive cells was determined by automated image analysis (**e**). Data are displayed as mean ± SEM of three independent experiments. ***p < 0.001 versus DMEM/F12 cultured pericytes (Student’s *t* test). Scale bar = 50 µm

To investigate basal phenotypic differences between D-pericytes and P-pericytes, cell morphology was investigated by brightfield microscopy at low confluence (3 days post-plating) and high confluence (7 days post-plating). Whilst D-pericytes displayed a disorganized cobblestone-like appearance with large cell bodies, P-pericytes appeared bipolar with cells growing in an aligned manner when confluent (Fig. 1a). Furthermore, P-pericytes displayed smaller cell nuclei (p < 0.001; Fig. 1b), significantly elevated cell numbers (p < 0.001; Fig. 1c) and an increased proliferative capability (p < 0.001; Fig. 1d, e) compared to D-pericytes.

### Culture media modifies expression of typical pericyte markers

**Fig. 2:**
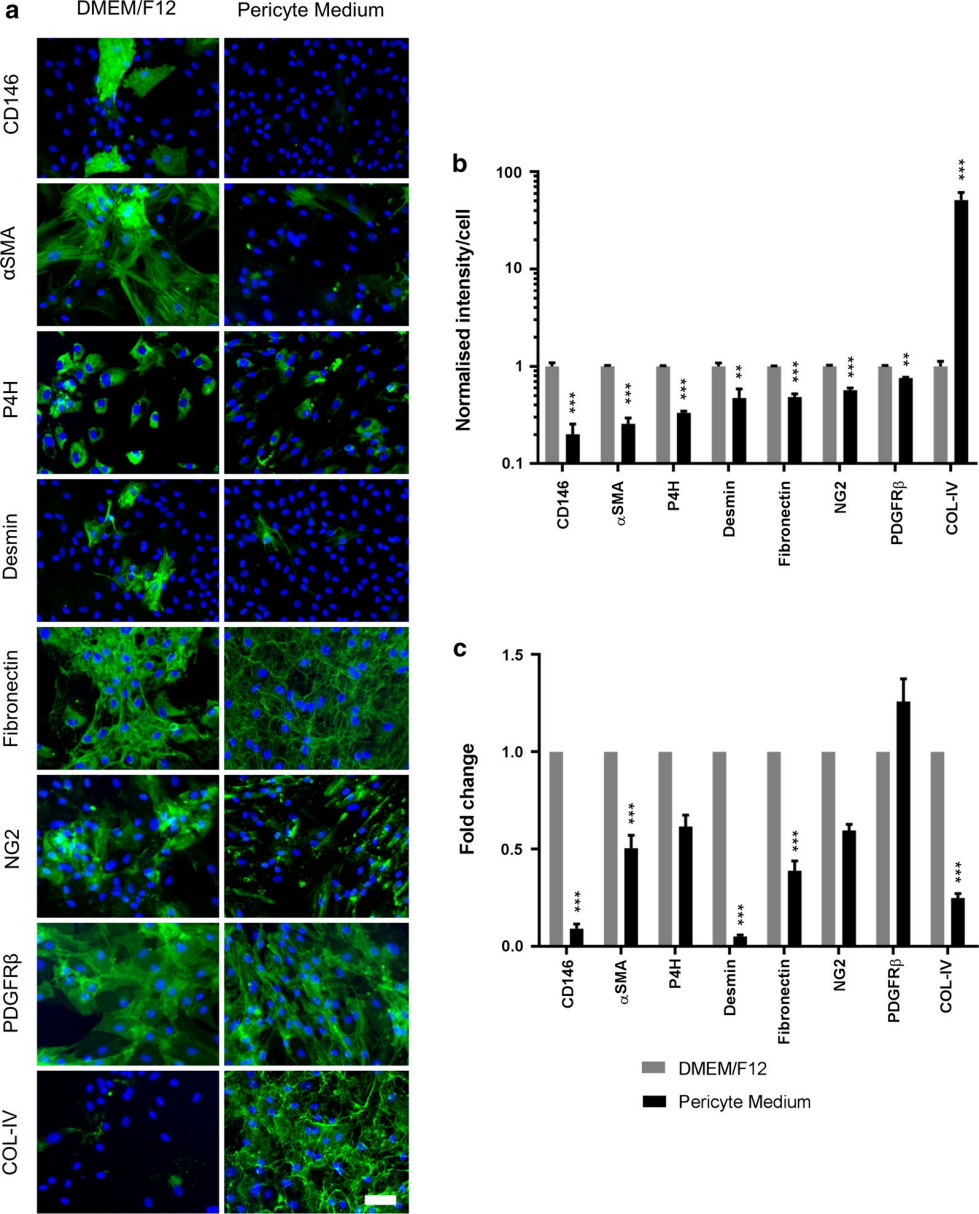
Culture media modifies expression of typical pericyte markers. Human brain pericytes were plated at a low density and allowed to proliferate in either DMEM/F12 with 10% FBS or Pericyte Medium for 7 days. At completion, pericytes were fixed and immunostained for the pericyte-associated markers CD146, αSMA, P4H, desmin, fibronectin, NG2, PDGFRβ and COL-IV. Nuclei were counterstained with Hoechst (**a**). The integrated intensity/cell of pericyte marker expression was determined by automated image analysis (**b**). RNA was extracted from samples treated as above and qRT-PCR performed to determine pericyte marker gene expression (**c**). Data are displayed as mean ± SEM of three independent experiments. **p < 0.01; ***p < 0.001 versus DMEM/F12 cultured pericytes (Student’s *t* test). Scale bar = 50 µm

In the absence of anatomical landmarks provided in vivo, the expression of pericyte markers is essential in characterising these cells under in vitro conditions. Having observed distinct pericyte morphologies between P-pericytes and D-pericytes we sought to investigate whether these translated to alterations in pericyte marker expression. Immunocytochemical analysis demonstrated that P-pericytes showed a significant reduction in several pericyte/fibroblast markers; most strikingly CD146 (p < 0.001) and αSMA (p < 0.001), but also P4H (p < 0.001), desmin (p < 0.01), fibronectin (p < 0.001), NG2 (p < 0.001) and PDGFRβ (p < 0.01; Fig. 2a, b). A significant increase in COL-IV was also seen in P-pericytes compared to D-pericytes (p < 0.001; Fig. 2a, b). Gene expression, as determined by qRT-PCR analysis, tended to correlate with protein expression, with a significant reduction in CD146 (*MCAM*; p < 0.001), αSMA (*ACTA2*; p < 0.001), desmin (*DES*; p < 0.001) and fibronectin (*FN1*; p < 0.001) and a trend towards attenuated P4H (*P4HA2*) and NG2 (*CSPG4*) expression in P-pericytes (Fig. 2c). However, PDGFRβ (*PDGFRB*) displayed a modest but non-significant increase (p > 0.05; Fig. 2c) whilst COL-IV (*COL4A1*) displayed a significant reduction in P-pericytes (p < 0.001; Fig. 2c).

### Culture media modifies IL-1β-induced transcription factor expression in human brain pericytes

**Fig. 3:**
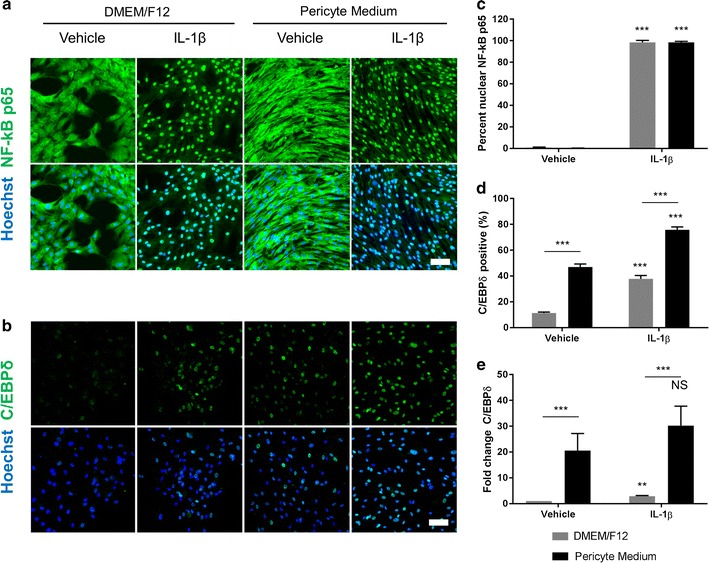
Culture media modifies IL-1β-induced transcription factor expression in human brain pericytes. Human brain pericytes were plated at a low density and allowed to proliferate in either DMEM/F12 with 10% FBS or Pericyte Medium for 7 days. For the final 1 h (**a**) or 24 h (**b**) 10 ng/mL IL-1β or vehicle was added. Pericytes were fixed and immunostained for the transcription factors NF-kB p65 (**a**) or C/EBPδ (**b**). Nuclei were counterstained with Hoechst. The percentage of cells displaying nuclear NF-kB p65 (**c**) or scored positive for C/EBPδ (**d**) was determined by automated image analysis. RNA was extracted from pericytes treated with 10 ng/mL IL-1β or vehicle (0.0001% BSA in PBS) for the final 24 h of a 7 day culture and the gene expression of *CEBPD* determined by qRT-PCR (**e**). Data are displayed as mean ± SEM of three independent experiments. NS = *p* > 0.05; **p < 0.01; ***p < 0.001 as designated or compared to vehicle control in respective media (Two-way ANOVA). Scale bar = 50 µm

Several studies have demonstrated a role for human brain pericytes in neuroinflammation [10, 20, 33, 39]. As master regulators of the immune response, transcription factors pose an attractive target to modify the expression of multiple inflammatory genes simultaneously. Both NF-kB and C/EBPδ have been previously studied with respect to modifying pericyte immune responses [10, 20, 39]. Under basal conditions, pericytes display cytoplasmic expression of NF-kB, irrespective of the media condition (Fig. 3a). Similarly, a 1 h stimulation with the pro-inflammatory cytokine IL-1β produces an almost complete nuclear translocation of NF-kB in both D-pericytes and P-pericytes (p < 0.001; Fig. 3a, c). Unlike NF-kB, C/EBPδ protein (p < 0.001; Fig. 3b, d) and gene expression (p < 0.001; Fig. 3e) was significantly higher in P-pericytes compared to D-pericytes. Whilst both D-pericytes and P-pericytes displayed elevated C/EBPδ protein expression after 24 h of IL-1β stimulation (D-pericytes p < 0.01, P-pericytes p < 0.001; Fig. 3b, d), the IL-1β-induced expression was significantly higher in P-pericytes than D-pericytes (p < 0.001; Fig. 3b, d). Elevated *CEBPD* gene expression was observed in D-pericytes after IL-1β treatment (p < 0.01; Fig. 3e), whilst P-pericytes displayed an elevated expression which was non-significant (p > 0.05; Fig. 3e). However, the IL-1β-induced gene expression of *CEBPD* was significantly higher in P-pericytes compared to D-pericytes (p < 0.001; Fig. 3e).

### Culture media modifies IL-1β-induced inflammatory mediator expression in human brain pericytes

**Fig. 4:**
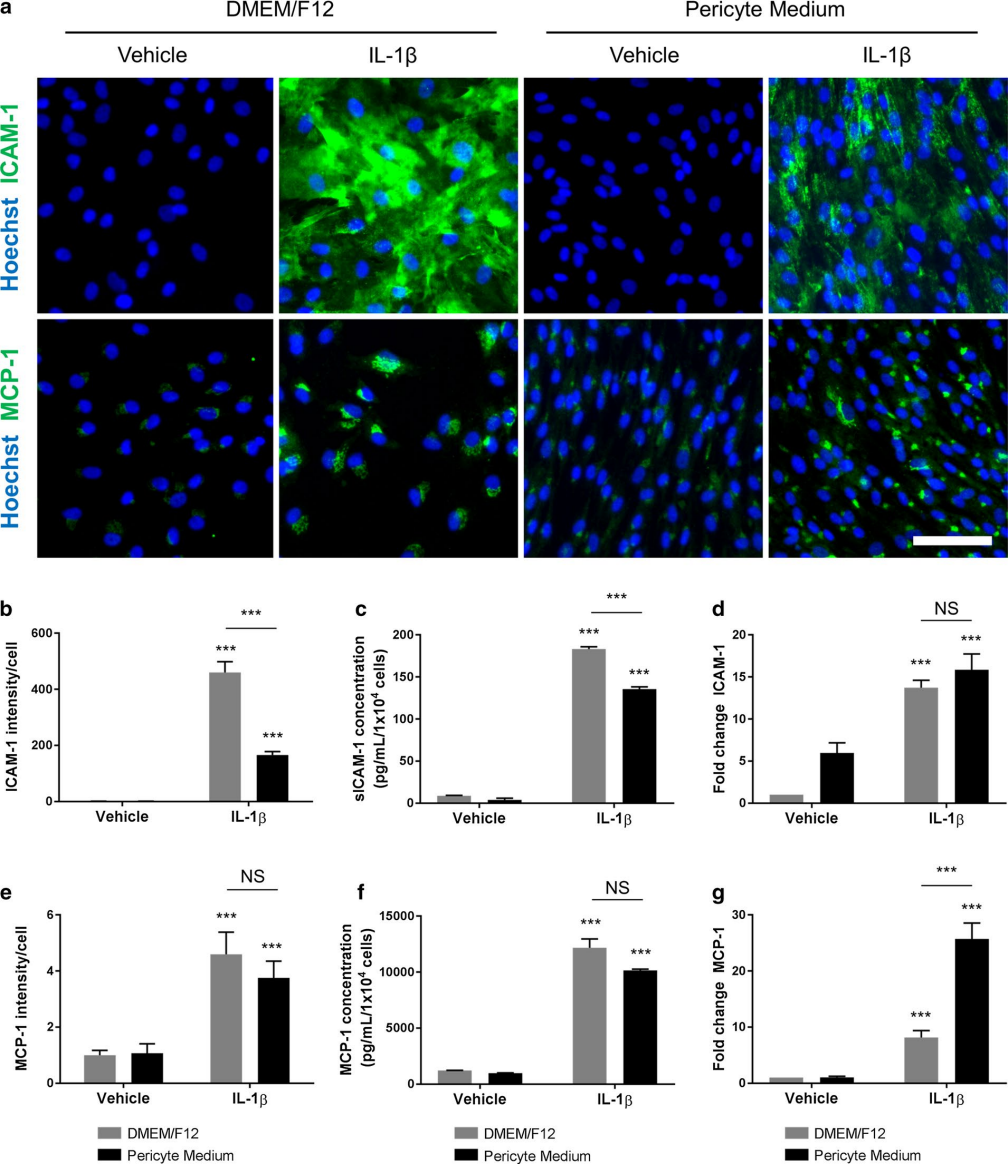
Culture media modifies IL-1β induced inflammatory mediator expression in human brain pericytes. Human brain pericytes were plated at a low density and allowed to proliferate in either DMEM/F12 with 10% FBS or Pericyte Medium for 7 days. For the final 24 h 10 ng/mL IL-1β or vehicle (0.0001% BSA in PBS) was added. Pericytes were fixed and immunostained for the inflammatory mediators ICAM-1 or MCP-1 (**a**). Nuclei were counterstained with Hoechst. The integrated intensity of ICAM-1/cell was determined by automated image analysis (**b**), the secretion of soluble ICAM-1 (sICAM-1) was determined by a cytometric bead array (**c**) and the gene expression of *ICAM1* was determined by qRT-PCR (**d**). Similarly, the integrated intensity of MCP-1/cell was determined by automated image analysis (**e**), the secretion of MCP-1 was determined by a cytometric bead array (**f**) and the gene expression of *MCP1* determined by qRT-PCR (**g**). Data are displayed as mean ± SEM from three independent experiments except CBA data which depicts mean ± SEM of triplicate wells from one representative case of three independent experiments. NS = p > 0.05; **p < 0.01; ***p < 0.001 as designated, or compared to vehicle control in respective media (Two-way ANOVA). Scale bar = 50 µm

C/EBPδ has been previously shown to modify the inflammatory response of human brain pericytes, particularly the expression of ICAM-1 and MCP-1 [39]. As C/EBPδ was significantly elevated in P-pericytes compared to D-pericytes, we sought to investigate whether this would correlate with functional changes in ICAM-1 and MCP-1 expression. Under basal conditions, ICAM-1 was found to be expressed at a very low level/absent in both cell conditions by immunocytochemistry and CBA (Fig. 4a–c). However, qRT-PCR analysis demonstrated that the gene transcript was significantly higher in P-pericytes (p < 0.01; Fig. 4d). Following a 24 h stimulation with IL-1β, ICAM-1 protein expression and sICAM-1 secretion was significantly elevated in both D-pericytes and P-pericytes (p < 0.001; Fig. 4a–d). However, the induction of both ICAM-1 protein expression, and sICAM-1 secretion was significantly lower in P-pericytes (p < 0.001; Fig. 4a–c). Interestingly, despite showing a higher basal expression, the IL-1β-induced ICAM-1 gene expression was not significantly different between D-pericytes and P-pericytes suggesting a reduction in the functional induction (p > 0.05; Fig. 4d). MCP-1 displays a modest basal expression and secretion in both culture conditions which were not significantly different from one another (p > 0.05; Fig. 4a, e–g). Like ICAM-1, MCP-1 expression and secretion was significantly increased following IL-1β stimulation in both D-pericytes and P-pericytes (p < 0.001; Fig. 4a, e–g); however the protein expression (p > 0.05; Fig. 4e) and secretion (p > 0.05; Fig. 4f) were not significantly different in either media condition. Interestingly, the gene expression of MCP-1 was significantly higher in P-pericytes compared to D-pericytes following IL-1β stimulation (p < 0.001; Fig. 4g).

### Culture media modifies phagocytic capacity of human brain pericytes

**Fig. 5:**
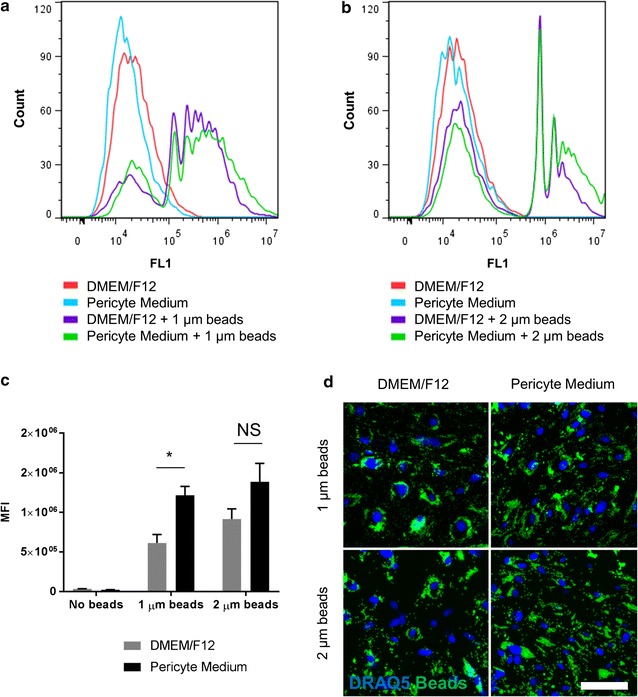
Culture media modifies phagocytic capacity of human brain pericytes. Human brain pericytes were plated at a low density and allowed to proliferate in either DMEM/F12 with 10% FBS or Pericyte Medium for 7 days. Cells were incubated with Fluoresbrite^®^ YG carboxylate microspheres of 1–2 µm diameter for 2 h and the phagocytic capacity of pericytes determined by flow cytometry. One representative plot for 1 µm beads (**a**) and 2 µm beads (**b**) is shown. The mean fluorescent intensity (MFI) from three independent experiments was determined (**c**). Pericytes were treated as above, except beads were incubated with cells for 24 h, fixed and nuclei counterstained with DRAQ5. Fluorescent microscopy images of one representative case from three independent experiments are shown (**d**). Data are displayed as mean ± SEM of three independent experiments. NS = *p* > 0.05; **p < 0.01; ***p < 0.001 (Student’s *t* test). Scale bar = 50 µm

Phagocytosis is an important aspect of the innate immune system and pericytes are able to phagocytose a range of particulate matter which may be beneficial in contributing to clearance of waste products from the CNS parenchyma [13–15]. Having shown that culture media can modulate inflammatory mediator expression, the ability of D-pericytes and P-pericytes to phagocytose mock waste products in the form of fluorescent beads was investigated. Following a 2 h incubation, both D-pericytes and P-pericytes phagocytosed 1–2 µm fluorescent beads as determined by flow cytometry (Fig. 5a–c). However, a significant increase in 1 µm bead uptake (p < 0.01; Fig. 5c) and a trend towards elevated uptake of 2 µm beads (p > 0.05; Fig. 5c) was observed in P-pericytes compared to D-pericytes. Internalisation and clustering of beads in the perinuclear region was evident after a 24 h incubation with beads in both media conditions (Fig. 5d).

### Culture media modifies pericyte migration following a scratch wound injury

**Fig. 6:**
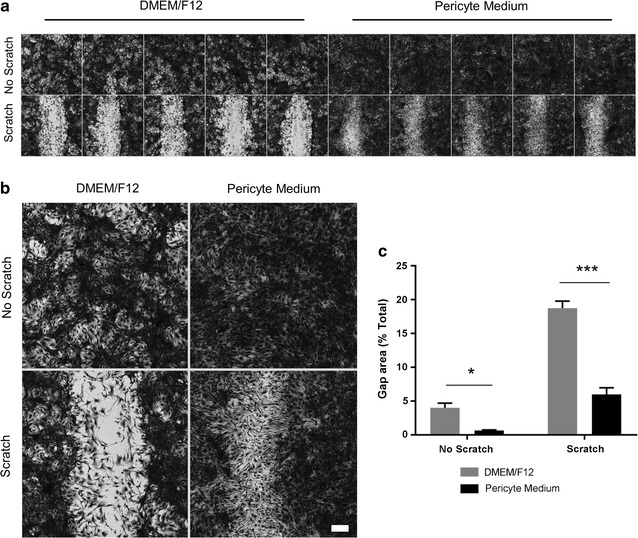
Culture media modifies pericyte migration following a scratch wound injury. Human brain pericytes were plated at a low density and allowed to proliferate in either DMEM/F12 with 10% FBS or Pericyte Medium for 7–14 days. The resulting confluent pericyte monolayer was scratched down the centre of the well using a sterile 200 µL pipette tip and wells were washed twice with PBS to remove detached cells. An equal number of wells were left unscratched. Pericytes were incubated for a further 48 h to allow migration into the scratch wound to occur. Cells were fixed and stained with Coomassie blue and imaged by bright field microscopy. Consistency of scratches between wells can be observed (**a**). A magnified image of a representative well shows the difference in pericyte migration (**b**). The gap area, defined as the percentage of each site devoid of Coomassie staining, was determined by automated image analysis (**c**). Data are displayed as mean ± SEM of three independent experiments. *p < 0.05; ***p < 0.001 (Two-way ANOVA with Bonferroni post-test). Scale bar = 50 µm

Pericyte migration is required during vasculogenesis, angiogenesis and non-glial cerebral scarring [4, 9, 42, 43]. Modulating pericyte migration may therefore be of interest and indeed has been studied with respect to several inflammatory cytokines and growth factors. We therefore sought to investigate whether culture media modified pericyte migratory potential. Pericytes were grown to full confluency and scratched down the centre of the well to form a gap into which pericytes can migrate. Scratches displayed excellent reproducibility between wells (Fig. 6a). A magnified view of representative wells demonstrates that in the absence of a scratch wound, P-pericytes were more confluent than D-pericytes as quantified by reduced area of coomassie blue staining (p < 0.001; Fig. 6b, c). Following a scratch wound injury, both D-pericytes and P-pericytes were able to migrate into the gap area, however, P-pericytes were significantly better at doing so (p < 0.001; Fig. 6b, c).

### Culture media does not alter pericyte responsiveness to growth factors TGFβ_1_ and PDGF-BB

**Fig. 7:**
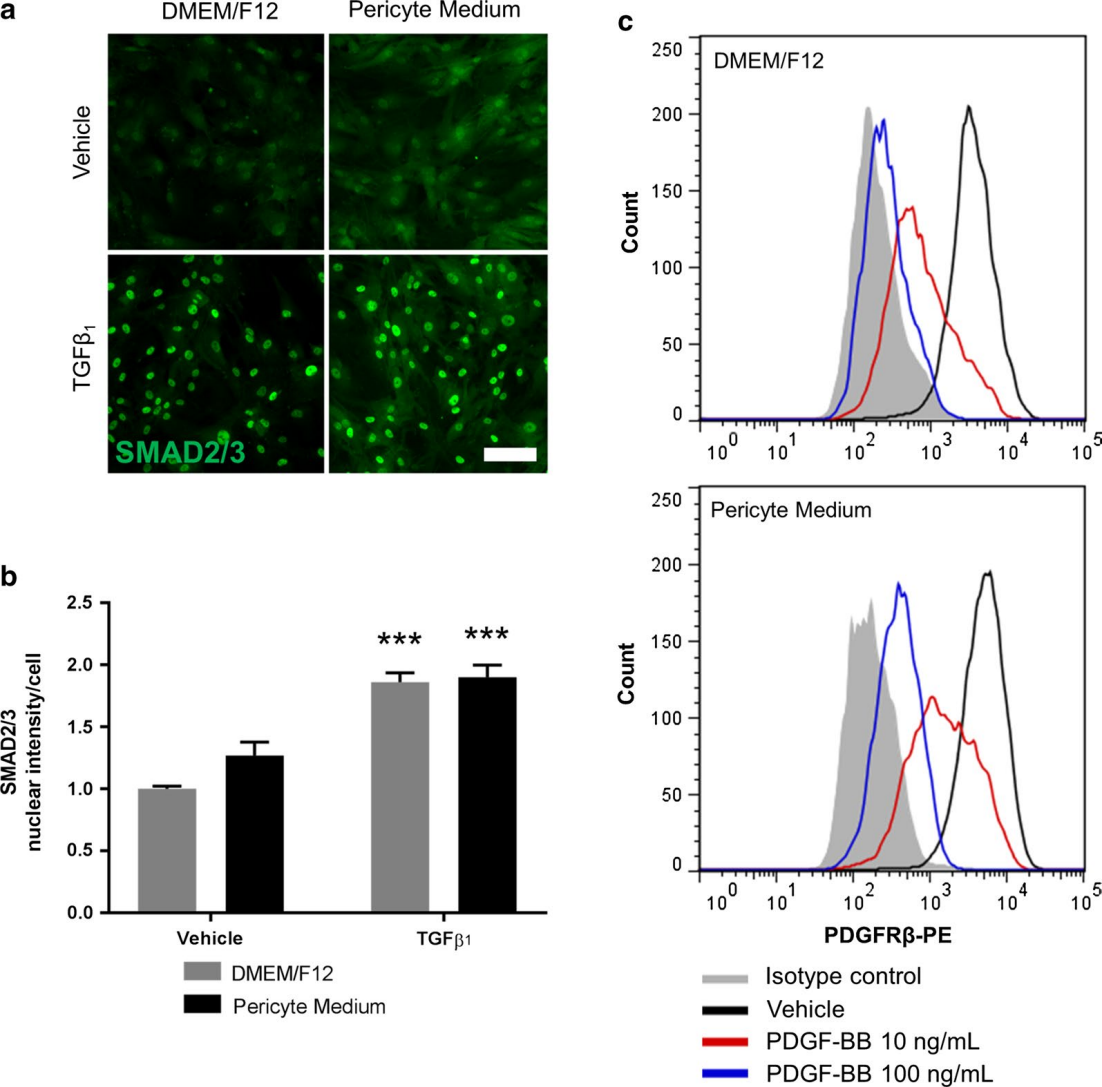
Culture medium does not alter pericyte responsiveness to growth factors TGFβ_1_ and PDGF-BB. Human brain pericytes were plated at a low density and allowed to proliferate in either DMEM/F12 with 10% FBS or Pericyte Medium for 7 days. For the final 2 h pericytes were incubated with 10 ng/mL TGFβ_1_ or vehicle (1 µM citric acid, pH 3 with 0.0001% BSA). Cells were fixed and immunostained for SMAD2/3 (**a**) and the nuclear intensity was determined by automated imagining analysis (**b**). Human brain pericytes were plated at a low density and allowed to proliferate in either DMEM/F12 with 10% FBS or Pericyte Medium for 7 days. For the final 30 min pericytes were treated with 10–100 ng/mL PDGF-BB or vehicle (4 µM hydrochloric acid with 0.0001% BSA). Cells were brought into suspension with Accutase and incubated with a PDGFRβ antibody or an isotype control. Cell surface PDGFRβ was determined by flow cytometry (**c**). Data are displayed as mean ± SEM of three independent experiments. ***p < 0.001 versus vehicle control in respective media (Two-way ANOVA). Scale bar = 50 µm

TGFβ_1_ and PDGF-BB are widely studied with respect to vascular biology and can modulate the pericyte phenotype and inflammatory responses [8, 28, 44–46]. To determine if D-pericytes and P-pericytes responded similarly to these growth factors we investigated their ability to induce nuclear SMAD2/3 induction in response to TGFβ_1_ and induce PDGFRβ internalisation in response to PDGF-BB, as previously demonstrated [45, 47]. Following a 2 h stimulation with TGFβ_1_, both D-pericytes and P-pericytes displayed elevated nuclear expression of SMAD2/3 (p < 0.001) which were not significantly different from one another (p > 0.05); Fig. 7a b). Similarly, both D-pericytes and P-pericytes displayed membrane PDGFRβ expression which was internalised following stimulation with PDGF-BB (Fig. 7c).

## Discussion

Growth conditions can have a major influence on the adopted phenotype and functional responses of cells cultured in vitro. Here we demonstrate that despite differences in their phenotype and proliferation rates, both D- and P-pericytes display similar migratory ability, phagocytic uptake, and growth factor and cytokine responses and therefore represent appropriate models to investigate neuroinflammatory responses.

Pericytes are a relatively poorly characterised cell type in the brain. Due to their similarities to several other cell types, particularly vascular smooth muscle cells and mesenchymal stem cells, the actual function and identification of pericytes in the literature varies significantly [8, 27]. Issues with pericyte characterisation in vitro are further exacerbated due to a lack of anatomical landmarks enabling pericyte identification. It is difficult then to determine which population (i.e., D-pericytes or P-pericytes) more accurately reflects that of pericytes seen in vivo. Several groups argue that true capillary-associated pericytes lack αSMA which is exclusive to vascular smooth muscle cells and myofibroblasts [6]. However, others have suggested that pericytes have a role in modulating cerebral blood flow, possibly through αSMA-mediated contractions [27]. Whilst desmin and CD146 are commonly used to identify pericytes, particularly in the mouse brain [28, 48], these proteins localise to large αSMA-positive vessels containing vascular smooth muscle cells in human brain tissue and were found to be lower in P-pericyte cultures (unpublished observations). Additionally, pericytes in vivo are often associated with extracellular collagen-IV expression which they secrete to aid basement membrane formation [49], and together these data suggest that P-pericytes more accurately recapitulate the in vivo pericyte phenotype. Whilst P-pericytes showed attenuated protein expression of the pericyte markers PDGFRβ and NG2 by image-based analysis, this could potentially be an artefact of the smaller cell size, and indeed the qRT-PCR data suggests no significant change in the expression of these pericyte markers.

Under basal conditions, both D-pericytes and P-pericytes display cytoplasmic localisation of NF-kB suggesting that neither culture condition sufficiently activated pericytes. Similarly, both populations showed complete nuclear translocation of NF-kB following IL-1β stimulation. Interestingly, P-pericytes showed a significantly greater basal and IL-1β-induced expression of C/EBPδ compared to D-pericytes. C/EBPδ is a transcription factor implicated in modifying neuroinflammatory responses, particularly in microglia and astrocytes [50–52]. Recently we have described a role for C/EBPδ in attenuating pericyte-derived ICAM-1 and MCP-1, both of which facilitate leucocyte infiltration into the brain [11, 12, 39, 53]. Interestingly, P-pericytes displayed a significantly blunted ability to induce ICAM-1, potentially due to enhanced C/EBPδ expression, although their induction of MCP-1 was not significantly reduced. Furthermore, both D-pericytes and P-pericytes were able to internalise fluorescent microparticles as an indicator of their phagocytic ability, although P-pericytes trended towards being more efficient phagocytes. Despite the differences in morphology and marker expression between D-pericytes and P-pericytes, both cultures maintained functional immune responses which differed only in the magnitude of change. Importantly, both culture conditions internalized these fluorescent microparticles at a rate much lower than that observed for professional macrophages in the form of primary human microglia [40].

Pericyte migration is essential during embryonic development, tumour progression, and cerebral scarring [4, 9, 43, 54]. As such, utilising in vitro models to understand how pericytes migrate is of interest. Whilst both D-pericytes and P-pericytes were found to efficiently migrate into a gap area following a scratch wound, P-pericytes did so at a significantly elevated rate. Importantly, this assay is likely to be influenced by the proliferative capacity of cells and P-pericytes display enhanced proliferation compared to D-pericytes. Furthermore, as a result of their elevated basal proliferation and smaller cell size, P-pericytes displayed greater confluency before scratch assays were performed which could also contribute to the enhanced rate of migration. Nevertheless, both D-pericytes and P-pericytes are feasible models for investigating drugs which modify pericyte migration and/or proliferation.

TGFβ_1_ and PDGF-BB are both widely studied with respect to vascular function and alter pericyte inflammatory responses in vitro [8, 27, 45, 46]. As distinct phenotypic differences were observed between D-pericytes and P-pericytes it is important to identify whether they respond differently to growth factor treatment. Utilising SMAD2/3 induction as a measure of TGFβ_1_ response and PDGFRβ internalisation as a measure of PDGF-BB response both P-pericytes and D-pericytes were able to respond to growth factor stimulation. However, determining the full response to TGFβ_1_ and PDGF-BB stimulation is warranted.

It is important to emphasise that human brain pericytes used in this study were cultured for four to six passages in DMEM/F12, 10% FBS and 1% PSG before being transferred into the defined Pericyte Medium for experimental analysis. The ability of pericytes to drastically alter their phenotype after being transferred into defined Pericyte Medium reflects and confirms the highly plastic nature of these cells.

Discrepancies between in vitro pericyte culture conditions and the data generated from these extends well beyond simple culture conditions (Additional file 1: Table S1). However, as the pericyte phenotype can be considerably modified by media composition, even when cultured from the same tissue for numerous passages, this raises questions about how other factors may influence in vitro and perhaps even in vivo data. A vital but often overlooked fact with respect to neuroinflammation is the vast differences between rodent immune cells and their human counterparts. This has been previously observed in the brain’s predominant immune cell the microglia [55], and more generally, inflammatory responses are poorly correlated between species [56]. Indeed, the current (admittedly limited) evidence suggests that non-human-derived pericytes display a significantly wider range of inflammatory mediators than human pericytes [57]. Rodent or porcine pericytes secrete several classical pro-inflammatory cytokines/mediators, including interferon-γ (IFNγ), tumour necrosis factor-α (TNFα), IL-1β and nitric oxide [14, 31, 32]. However, these mediators are not found to be secreted from human pericyte cultures, although they do secrete several other inflammatory factors including IL-6, IL-8, and MCP-1 [20, 39]. There is less direct evidence for the involvement of other variables including age and the isolation procedure; however, like culture medium and donor species, these differences could potentially influence the pericyte phenotype and functional responses.

During normal aging [58] and several pathological conditions including stroke [59], traumatic head [60] and spinal injuries [61], and in neurodegenerative disorders such as Alzheimer’s disease [62] and amyotrophic lateral sclerosis [63], there is significant disruption of the BBB and the blood–spinal cord barriers. Under these conditions, serum from blood enters the brain and spinal cord and although not well characterised this is likely to have profound effects on brain and spinal cord cells, including pericytes. Our data shows that human brain-derived pericytes cultured in high serum conditions (as observed in the D-media) show altered responses, although not completely different, to the P-media conditions. Of importance, in these experiments fetal calf serum was used and it is plausible that human serum might produce a vastly different phenotype.

## Conclusions

 Whilst several functional outcomes differed in terms of absolute values, it is important to emphasise that the actual neuroinflammatory responses between D-pericytes and P-pericytes were not dissimilar. Cells grown in both conditions mounted a functional immune response including NF-kB translocation, C/EBPδ induction, ICAM-1/MCP-1 induction and secretion, and phagocytosis. Furthermore, both D-pericytes and P-pericytes maintained proliferative capability and migrated after a scratch wound injury and responded to TGFβ_1_ and PDGF-BB treatment. It therefore appears that whilst certain differences should be considered, both D-pericytes and P-pericytes represent appropriate models to study functional pericyte neuroinflammatory responses.

## Additional files

**Additional file 1: Table S1.** In vitro culture conditions of brain pericytes in selected publications. This table lists previous publications describing in vitro pericyte cell culture conditions.

**Additional file 2: Table S2.** List of BD Biosciences Flex Sets used for CBA. This table lists the BD Biosciences Flex Sets used for the CBA studies.

**Additional file 3: Table S3.** List of antibodies used for ICC and flow cytometry. This table provides a list of antibodies used for the ICC and flow cytometry studies.

**Additional file 4: Table S4.** List of primers used for qRT-PCR. This table lists the primers used for the qRT-PCR studies.
