# Supplementary material for: Modelling physiological and pathological conditions to study pericyte biology in brain function and dysfunction
Source: BMC Neurosci. 2018 Feb 22;19:6. doi: 10.1186/s12868-018-0405-4 (PMC5824614; doi:10.1186/s12868-018-0405-4)
Supplement: Supplementary file 1 — Additional file 1: Table S1. In vitro culture conditions of brain pericytes in selected publications. This table lists previous publications describing in vitro pericyte cell culture conditions. [file 12868_2018_405_MOESM1_ESM.docx]

**Table S1: *In vitro* culture conditions of brain pericytes in selected publications.**

| Reference | Species | Age | Isolation procedure | Culture media | Study scope |
| --- | --- | --- | --- | --- | --- |
| [[10](#_ENREF_10)] | Human | Adult | Whole tissue | DMEM/F12, 10% FBS, 1% PSG | Inflammation |
| [[30](#_ENREF_30)] | Human | N/A | N/A (ScienCell, Ca, USA) | Pericyte Medium (ScienCell | Inflammation |
| [[31](#_ENREF_31)] | Mouse | N/A | Microvessel | DMEM/F12, 20% FCS, 2 mM L-Glutamine, gentamicin | Inflammation |
| [[20](#_ENREF_20)] | Human | N/A | N/A (ScienCell) | Pericyte Medium (ScienCell) | Inflammation |
| [[32](#_ENREF_32)] | Rat | Infant | Whole tissue | DMEM, 20% FBS | Inflammation |
| [[33](#_ENREF_33)] | Human | N/A | N/A (Cell Systems, WA, USA) | Pericyte Medium (ScienCell) | Inflammation |
| [[14](#_ENREF_14)] | Pig | Adult | Whole tissue | DMEM/F12, 10% serum, 1% PSG, 1% gentamycin | Inflammation/ Phagocytosis |
| [[13](#_ENREF_13)] | Rat | 6-8w | Microvessel | DMEM/F12, 10% FBS, 1% PSG | Phagocytosis |
| [[34](#_ENREF_34)] | Mouse | 1-9m | Microvessel | DMEM, 10% FBS, 1% non-essential amino acids, 1% vitamins, 1% antibiotic/mycotic | Aβ internalisation |
| [[35](#_ENREF_35)] | Mouse | 8w | Whole tissue | Pericyte Medium (ScienCell) | Migration/ Proliferation |
| [[36](#_ENREF_36)] | Rat | 3w | Microvessel | DMEM, 20% FBS, 50 µg/ml gentamicin | Migration |
