# Supplementary material for: Modelling physiological and pathological conditions to study pericyte biology in brain function and dysfunction
Source: BMC Neurosci. 2018 Feb 22;19:6. doi: 10.1186/s12868-018-0405-4 (PMC5824614; doi:10.1186/s12868-018-0405-4)
Supplement: Supplementary file 2 — Additional file 2: Table S2. List of BD Biosciences Flex Sets used for CBA. This table lists the BD Biosciences Flex Sets used for the CBA studies. [file 12868_2018_405_MOESM2_ESM.docx]

**Table S2: List of BD Biosciences Flex Sets used for CBA**

| Antibody | Catalogue # | Bead position |
| --- | --- | --- |
| sCD54/ICAM-1 | 560269 | A4 |
| MCP-1 | 558287 | D8 |
