# Supplementary material for: Modelling physiological and pathological conditions to study pericyte biology in brain function and dysfunction
Source: BMC Neurosci. 2018 Feb 22;19:6. doi: 10.1186/s12868-018-0405-4 (PMC5824614; doi:10.1186/s12868-018-0405-4)
Supplement: Supplementary file 3 — Additional file 3: Table S3. List of antibodies used for ICC and flow cytometry. This table provides a list of antibodies used for the ICC and flow cytometry studies. [file 12868_2018_405_MOESM3_ESM.docx]

**Table S3: List of antibodies used for ICC and flow cytometry**

| Antibody | Company | Catalogue # | Dilution |
| --- | --- | --- | --- |
| Mouse anti-C/EBPδ | Santa Cruz | sc-365546 | 1:250 |
| Mouse anti-Collagen IV | Dako | M6785 | 1:500 |
| Mouse anti-NG2 | Santa Cruz | sc-53389 | 1:500 |
| Rabbit anti-NF-kB p65 | Santa Cruz | sc-372 | 1:500 |
| Mouse anti-ICAM-1 | Santa Cruz | sc-107 | 1:500 |
| Rabbit anti-αSMA | Abcam | ab5694 | 1:100 |
| Rabbit anti-Fibronectin | DAKO | A0245 | 1:10,000 |
| Rabbit anti-PDGFRβ | Cell Signalling | mAb3169 | 1:500 |
| Rabbit anti-P4H | Sigma | HPA0075991 | 1:500 |
| Rabbit anti-MCP1 | Abcam | ab74121 | 1:500 |
| Mouse anti-SMAD2/3 | Santa Cruz | sc-133098 | 1:500 |
| Mouse anti-CD146 | Abcam | ab24577 | 1:500 |
| Rabbit anti-Desmin | Abcam | ab53765 | 1:500 |
| Goat anti-mouse Alexa Fluor® 488 | Invitrogen | A11001 | 1:500 |
| Goat anti-mouse Alexa Fluor® 594 | Invitrogen | A11005 | 1:500 |
| Goat anti-rabbit Alexa Fluor® 488 | Invitrogen | A11008 | 1:500 |
| Goat anti-rabbit Alexa Fluor® 594 | Invitrogen | A11012 | 1:500 |
| Mouse anti-CD140b-PE | BD Biosciences | 558821 | 1:6.25 (flow) |
| Mouse IgG2a, κ isotype control-PE | BD Biosciences | 555574 | 1:6.25 (flow) |
