# Supplementary material for: Modelling physiological and pathological conditions to study pericyte biology in brain function and dysfunction
Source: BMC Neurosci. 2018 Feb 22;19:6. doi: 10.1186/s12868-018-0405-4 (PMC5824614; doi:10.1186/s12868-018-0405-4)
Supplement: Supplementary file 4 — Additional file 4: Table S4. List of primers used for qRT-PCR. This table lists the primers used for the qRT-PCR studies. [file 12868_2018_405_MOESM4_ESM.docx]

**Table S4: List of primers used for qRT-PCR**

| Gene | Protein |  | Sequence | Amplicon Length |
| --- | --- | --- | --- | --- |
| *GAPDH* | GAPDH | Fw | CATGAGAAGTATGACAACAGCCT | 113 bp |
|  |  | Rv | AGTCCTTCCACGATACCAAAGT |  |
| *ICAM1* | ICAM1 | Fw | GAACCAGAGCCAGGAGACAC | 84 bp |
|  |  | Rv | GAGACCTCTGGCTTCGTCAG |  |
| *IL6* | IL-6 | Fw | TTCGGTCCAGTTGCCTTCTC | 80 bp |
|  |  | Rv | TCTTCTCCTGGGGGTACTGG |  |
| *IL6* | IL-8 | Fw | CAGAGACAGCAGAGCACACA | 70 bp |
|  |  | Rv | GTGAGATGGTTCCTTCCGGT |  |
| *CEBPD* | C/EBPδ | Fw | TTCAGCGCCTACATCGACTC | 80 bp |
|  |  | Rv | TTGAAGAGGTCGGCGAAGAG |  |
| *MCP1* | MCP-1 | Fw | CAGCCAGATGCAATCAATGCC | 190 bp |
|  |  | Rv | TGGAATCCTGAACCCACTTCT |  |
| *COL4A1* | COL-IV | Fw | CCCGAAAGGCCAGCAAGGTGTT | 77 bp |
|  |  | Rv | GGGCACCGTCAAACCCAGGAAT |  |
| *ACTA2* | αSMA | Fw | ACGTGGGTGACGAAGCACAGA | 84 bp |
|  |  | Rv | CGTCCCAGTTGGTGATGATGCC |  |
| *PDGFRB* | PDGFRβ | Fw | CGCAAAGAAAGTGGGCGGCT | 80 bp |
|  |  | Rv | TGCAGGATGGAGCGGATGTGGT |  |
| *P4HA2* | P4H | Fw | TCCAGTTGGGTGATCTGCACCG | 77 bp |
|  |  | Rv | AGCTCGTTCGTGGCTTGGGT |  |
| *CSPG4* | NG2 | Fw | ATGGGAGGTTGGCTTGGCGT | 81 bp |
|  |  | Rv | GGCCACGCAACAGGTCTTCA |  |
| *FN1* | Fibronectin | Fw | CGAGAGTGCCCCTACTACAC | 84 bp |
|  |  | Rv | TGTTGGTGAATCGCAGGTCA |  |
| *DES* | Desmin | Fw | TTGACCTGGAGCGCAGAATT | 84 bp |
|  |  | Rv | GCAACTCACGGATCTCCTCTT |  |
| *MCAM* | CD146 | Fw | AGTCCCAAGGCAACCTCAGCCA | 76 bp |
|  |  | Rv | CGCACACGGAAGATGAGCGT |  |
